# Epigenetic Inheritance, Epimutation, and the Response to Selection

**DOI:** 10.1371/journal.pone.0101559

**Published:** 2014-07-14

**Authors:** Robert E. Furrow

**Affiliations:** Department of Biology, Stanford University, Stanford, California, United States of America; Tel Aviv University, Israel

## Abstract

There has been minimal theoretical exploration of the role of epigenetic variation in the response to natural selection. Using a population genetic model, I derive formulae that characterize the response of epigenetic variation to selection over multiple generations. Unlike genetic models in which mutation rates are assumed to be low relative to the strength of selection, the response to selection decays quickly due to a rapid lowering of parent-offspring epiallelic correlation. This effect is separate from the slowing response caused by a reduction in epigenetic variation. These results suggest that epigenetic variation may be less responsive to natural selection than is genetic variation, even in cases where levels of heritability appear similar.

## Introduction

Although epigenetic variation has been observed in many wild populations [1]–[6] and can be inherited across meiotic generations [7]–[18], its role in phenotypic heritability and adaptive evolution is unclear. Theoreticians and empiricists often hint at the potential importance of epigenetic processes in adaptation [19], [20], but Holeski *et al.* [21] note that “… no multigenerational experiments have evaluated the relative contribution of epigenetic inheritance in response to natural selection.” Despite this, recent results suggest that epigenetic variation can play a role in adaptation. Cropley *et al.* [22] selected for coat color on mice with induced epigenetic variability and found that the methylation-associated phenotype increased progressively over generations, as long as a dietary generator of epigenetic variation was present. In a review of epigenetic variation and inheritance in plants, Hirsch *et al.* [23] share the results of a currently unpublished selection experiment using *Arabidopsis thaliana*, in which a selected line and its genetically identical ancestor consistently differed in phenotype and in cytosine methylation status. In both of these systems, it appears that epigenetic variation responded to selective pressures.

Current population genetic theory doesn't readily lend itself to an intuitive or analytical understanding of such an adaptive response over multiple generations. Day and Bonduriansky [24] present a very nice theoretical analysis of the transgenerational change in phenotype and genotype frequencies for an array of non-genetic inheritance models, but only derive formulae for change over a single generation. Geoghegan and Spencer [25] explore the properties of evolutionarily stable equilibria when mutation rates are environment-sensitive. However, they focus primarily on the characteristics of the equilibria as opposed to the dynamics of the adaptive response. Klironomos *et al.* [26] use simulation modeling to understand the response to selection on a fitness landscape where the phenotypic optimum can be achieved by either genetic or epigenetic variants. They find that epigenetic adaptation may occur rapidly as a transient process before genetic adaptation ultimately supersedes, although the authors do not analyze the rate of this process.

To develop an intuitive understanding of epigenetic variation's role in sustained adaptive evolution, I derive analytical formulae to characterize the response to selection at an epigenetic locus over multiple generations. For simplicity, I focus on one of the simplest models of epigenetic inheritance: the case where the environment is homogeneous, epimutation rates are constant, and viability selection acts on variation at a single epigenetic locus. Unlike genetic mutation, rates of epimutation are poorly understood and may not be orders of magnitude lower than the strength of selection [18], [27], so my analytical approximation does not ignore higher order mutation terms. The results suggest that higher potential mutation rates of epigenetic marks can lead to a rapid decay in the response to selection over multiple generations, with this response increasingly reduced in successive generations of selection.

## Model

**Figure 1:**
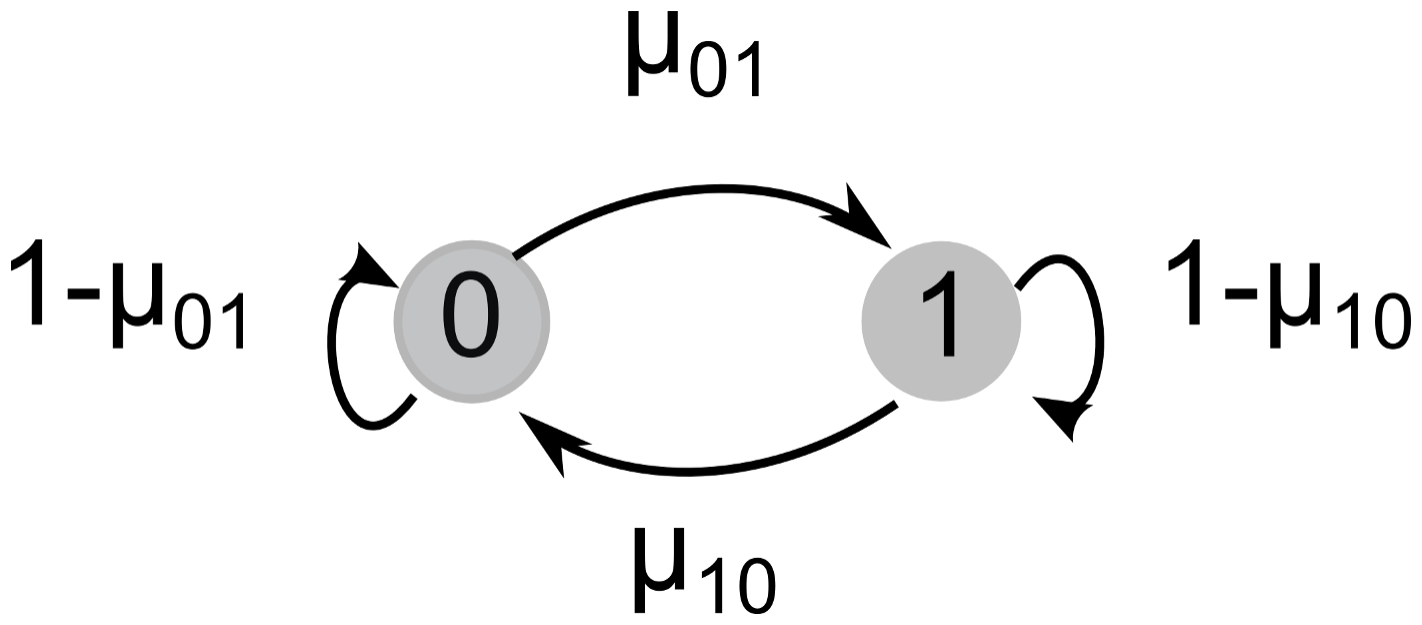
Epimutation rates. Between generations, an allele mutates between states with probabilities shown above.

I consider a dynamic haploid model identical to that of Slatkin [28], where an epiallele can take one of two states, 0 or 1, in a particular generation. The individual contributes a fraction of offspring to the next generation that is proportional to its relative fitness. The distribution of states of an individual's offspring are determined by the epimutation rates 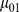 and 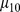 (Figure 1). I define the parameter 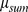 to be the sum of epimutation rates, 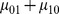, and assume that state 0 has fitness lower than state 1 by a factor 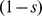. Table 1 summarizes the epigenetic states, frequencies, and fitness values.

**Table 1:** Epialleles, epiallele frequencies, and their fitnesses.

allele	freq.	fitness
1	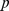	1
0	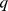	1-s

In a large population with a life cycle of selection, epimutation, and reproduction, the frequency of epiallele 1 in the next generation, 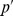, can be written in terms of the epiallele frequencies in the current generation as
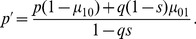
(1)


Note that a diploid model in which the viability fitnesses of the heterozygote and the 00 homozygote are equal to 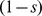 and 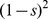, respectively, will produce an identical recursion in epiallele frequency. Thus the results presented here extend to the diploid case where the two epialleles within an individual mutate and affect fitness independently of one another.

## Results

### Epiallelic correlation

Consider the haploid population with epigenetic variation at a locus. I define 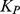 and 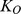 to be random variables for the epigenetic state of a random parent and one of their offspring. I assume that the population begins at the neutral epimutational equilibrium where the frequency of epiallele 1 is 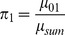, and the frequency of epiallele 0 is 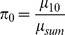, where 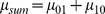. In this case, the covariance between parent and offspring is
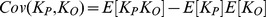


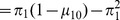


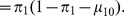



The variances of these two variables at neutral equilibrium are identical and equal to
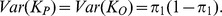



Combining these formulae, the parent-offspring correlation in epiallelic state is
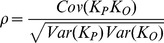


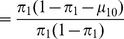


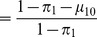


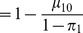


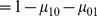


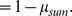



### Exact equilibrium equation

Equation (1) represents a linear fractional system with strictly positive terms, so it will converge to the unique stable equilibrium given by the larger solution to the quadratic equation defined when 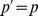. The solution is
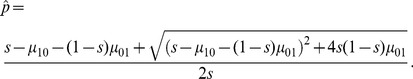
(2)


Because this solution does not readily lend intuition to the rate of adaptation nor to the relative roles of variance, selection strength, and mutation rate, the following results focus on an approximation to equation (1).

### Approximate recursions for population response to selection

The exact recursion for the change in allele frequencies, equation (1), can be re-arranged as
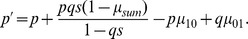



Assuming that the selection coefficient 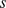 is relatively small, the recursion can be approximated by a Taylor expansion of equation (1) in which terms of order 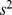 and higher are ignored. This yields
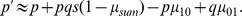



To assess the longer term response to selection, I assume that the initial allele frequencies in the population are at epimutation balance, where 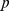 at time 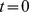 is equal to 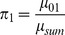. From these initial conditions, the neutral mutational terms in the exact equation cancel each other.
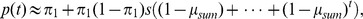
and
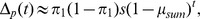
where 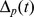 is the change in allele frequency between generation 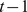 and generation 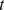.

The approximate response to selection is proportional to three factors: the initial epiallelic variance 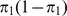, the strength of selection 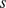, and a power of the initial parent-offspring epiallelic correlation 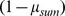. Because this final factor is raised to the 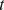-th power, the exact epiallelic correlation will crucially determine whether there is a sustained response to selection. Given this recursion, the approximate equilibrium frequency of allele 1 at epimutation-selection balance is
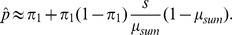



**Figure 2:**
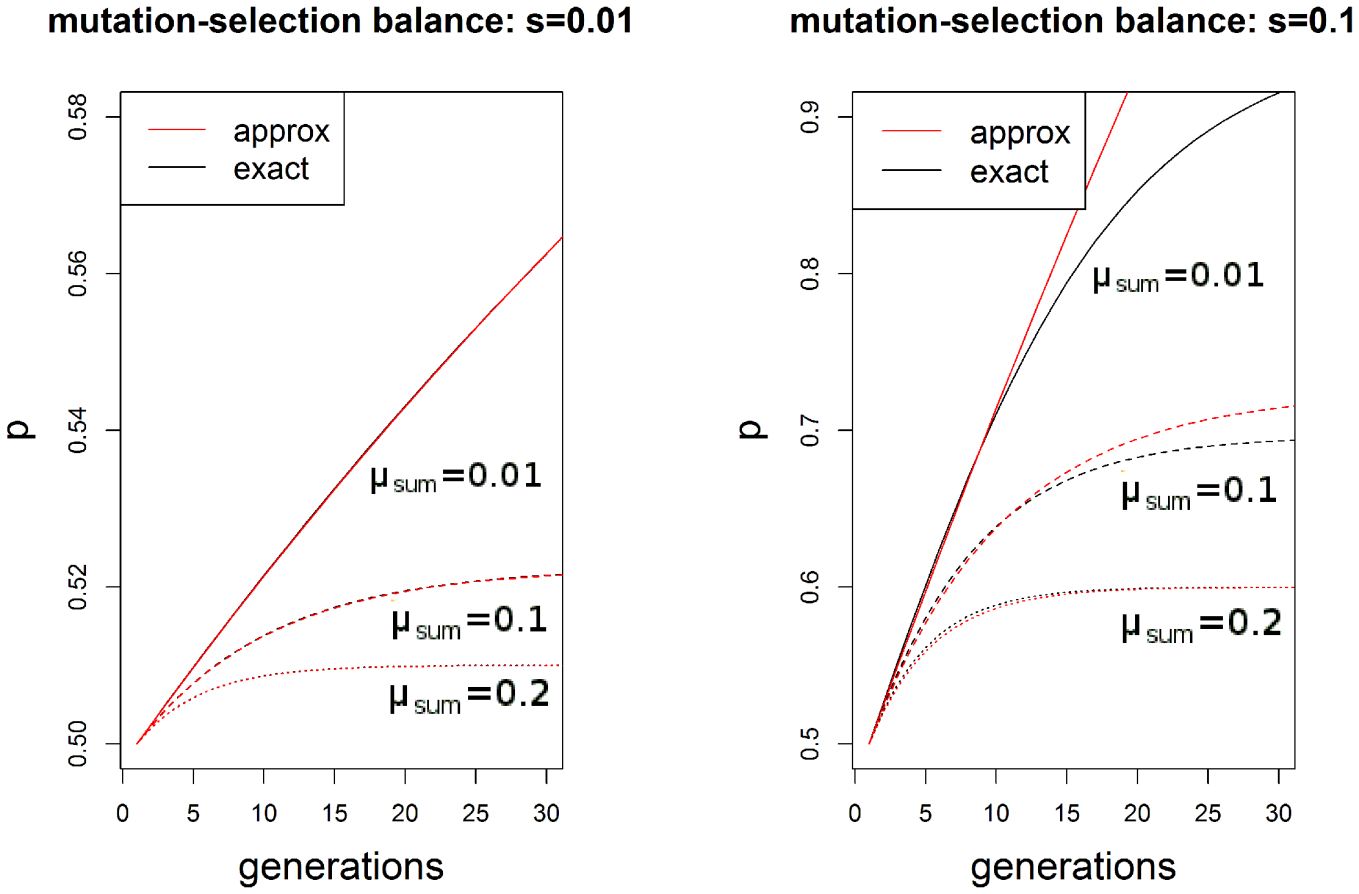
Exact and approximate approaches to epimutation-selection balance. Selection is weaker in the left panel (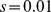) than the right panel (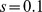). The horizontal axis in both panels represents the number of generations since selection began, and the vertical axis represents the epiallele frequency of the advantageous epiallele 1. Red lines correspond to approximate trajectories, and black lines are the exact trajectories found by simulation. In the left panel, the approximation closely matches the exact results, making the red and black lines indistinguishable. The type of dashes indicate the value of 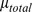.

As mutation rates increase, the response to selection is weaker and there is more rapid convergence to the equilibrium (Figure 2). The approximation holds well as long as the strength of selection 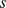 is smaller than 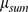. Alternatively, this approximate equilibrium can be derived from the exact equilibrium equation (2) by Taylor expanding around 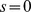 and ignoring terms of 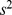 and higher. Because equation (1) also represents a diploid population in which the two epialleles act independently, these results apply in that case as well.

## Discussion

In the absence of selection, non-environmentally-sensitive epigenetic variation contributes to parent-offspring phenotypic covariance in a manner nearly identical to that of genetic variation. There is simply one additional discounting factor 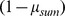, which corresponds to the allelic correlation between parent and offspring at an epigenetic locus in a haploid population. This is analagous to the factor 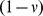 presented by Tal *et al.* [29]. Under selection, the correlation factor 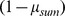 characterizes the rate of decay of the response, and also plays a role in determining the epiallele frequencies at epimutation-selection balance. Because the factor 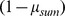 is taken to progressively higher powers in subsequent generations of selection, an epigenetically controlled phenotype may demonstrate a reduced response to selection in comparison to a genetically controlled phenotype, even if the initial contributions to phenotypic heritability are similar.

In practice, this effect should be observable in an artificial selection experiment. Johannes *et al.* [13] performed a single-generation selection experiment with epigenetically variable lines. An extension of this work over multiple generations should shed light on these theoretical results, and help characterize responses to natural selection in wild populations. However, as opposed to the constant epimutation rates modeled here, environmentally-sensitive epimutation rates may yield a less predictable selective response. If the selective pressures also influence the rates of epimutation, mutational processes may serve to either magnify the reductive effect or counter it, depending on how the rates of epimutation are affected. Genetic variation may also interact with epigenetic variation in the response to selection. One possibility is that epigenetic variation plays a role in the initial response to selection, while genetic variation shifts more slowly but ultimately produces a phenotypic distribution in the population with less mutational load [26]. Further empirical and theoretical research on the epigenetic response to selection should help clarify how natural populations will respond to novel selective pressures: an issue of great concern as humans continue to rapidly modify landscapes around the world.
